# A Novel Inflammatory Response–Related Gene Signature Improves High-Risk Survival Prediction in Patients With Head and Neck Squamous Cell Carcinoma

**DOI:** 10.3389/fgene.2022.767166

**Published:** 2022-04-11

**Authors:** Yanxun Han, Zhao Ding, Bangjie Chen, Yuchen Liu, Yehai Liu

**Affiliations:** ^1^ Department of Otorhinolaryngology, Head and Neck Surgery, The First Affiliated Hospital of Anhui Medical University, Hefei, China; ^2^ Anhui Medical University, Hefei, China; ^3^ Graduate School of Anhui Medical University, Hefei, China; ^4^ The First Affiliated Hospital of Anhui Medical University, Hefei, China

**Keywords:** head and neck squamous cell carcinoma, inflammatory response, prognosis, chemotherapy sensitivity, immune cell infiltration

## Abstract

**Background:** Head and neck squamous cell carcinoma (HNSCC) is a highly prevalent and malignant tumor that is difficult to effectively prognosticate outcomes. Recent reports have suggested that inflammation is strongly related to tumor progression, and several biomarkers linked to inflammation have been demonstrated to be useful for making a prognosis. The goal of this research was to explore the relevance between the inflammatory-related genes and HNSCC prognosis.

**Methods:** The clinical information and gene expression data of patients with HNSCC were acquired from publicly available data sources. A multigene prognostic signature model was constructed in The Cancer Genome Atlas and verified in the Gene Expression Omnibus database. According to the risk score calculated for each patient, they were divided into low- and high-risk groups based on the median. The Kaplan–Meier survival curve and receiver operating characteristic curve were applied to determine the prognostic value of the risk model. Further analysis identified the independent prognostic factors, and a prognostic nomogram was built. The relationship between tumor immune infiltration status and risk scores was investigated using Spearman correlation analysis. Finally, to confirm the expression of genes in HNSCC, quantitative real-time polymerase chain reaction (qRT-PCR) was performed.

**Results:** A prognostic model consisting of 14 inflammatory-related genes was constructed. The samples with a high risk had an apparently shorter overall survival than those with a low risk. Independent prognostic analysis found that risk scores were a separate prognostic factor in HNSCC patients. Immune infiltration analysis suggested that the abundance of B cells, CD8 T cells, M2 macrophages, myeloid dendritic cells, and monocytes in the low-risk group was higher, while that of M0, M1 macrophages, and resting NK cells was obviously higher in the high-risk group. The risk scores were related to chemotherapeutic sensitivity and the expression of several immune checkpoint genes. Moreover, CCL22 and IL10 were significantly higher in HNSCC tissues, as determined by qRT-PCR.

**Conclusion:** Taken together, we constructed a novel inflammatory response–related gene signature, which may be used to estimate outcomes for patients with HNSCC and may be developed into a powerful tool for forecasting the efficacy of immunotherapeutic and chemotherapeutic drugs for HNSCC.

## Introduction

Head and neck cancers (HNCs) are a group of heterogeneous diseases and include cancers occurring in the tongue, mouth, pharynx, and larynx and other oral cavities (Haddad and Shin, 2008). HNC ranks sixth in terms of the global cancer incidence rate and accounts for about 4% of all cancers. In the United States in 2020, it was estimated that HNC accounted for about 65,630 new oral cavity, pharynx, and larynx cancer cases and 14,500 deaths (Ferlay et al., 2019; Siegel et al., 2020). According to histopathologic classification, about 90% of HNCs are squamous cell carcinoma (Ma et al., 2020). The specific etiology of head and neck squamous cell carcinoma (HNSCC) is unknown. Previous studies have suggested that genetic factors, smoking, alcohol consumption, and viral infections [e.g., Epstein–Barr virus (EBV) and human papillomavirus (HPV)] were the most common risk factors (Mehanna et al., 2013; Maasland et al., 2014; Turunen et al., 2017). Surgery, radiation, chemotherapy, and molecular targeted medicines are all options for HNSCC treatment. Although there has been an improvement in the therapeutic modalities available, the clinical prognosis has not evolved significantly. Due to the high incidence of local recurrence and distant metastases, the 5-year survival rate of HNSCC patients is lower than 50% (Chow, 2020). Identification of novel molecular biomarkers is significant for improving the prognosis of these patients because of the multiple anatomical locations, molecular heterogeneity, and diverse etiology of HNSCC.

Since inflammation may contribute to the development of various cancers, the biological processes between inflammation and carcinogenesis have been the focus of research (Greten and Grivennikov, 2019). Chronic inflammation induces tumor angiogenesis and DNA damage, and promotes tumor proliferation and metastasis while preventing apoptosis (Grivennikov et al., 2010). Some studies have revealed a partial association between cancer and inflammatory markers by analyzing peripheral blood–related parameters. For example, many inflammatory response features like leukocytosis, hypoproteinemia, and hyperfibrinogenemia were confirmed in HNSCC patients (Cai et al., 2020; Zhou et al., 2019). In recent years, the use of systemic hematology inflammatory markers as prognostic factors in malignancy has attracted increasing attention. Neutrophil, lymphocyte, monocyte, and platelet counts, both individually and expressed as ratios, showed significant overall survival (OS) prognostic ability and were independent of existing prognostic factors for HNSCC (Zhou et al., 2020; Rassouli et al., 2015). Mcmillan reported that the Glasgow Prognostic Score, consisting of peripheral blood inflammatory markers, was an independent prognostic factor in cancer patients (Mcmillan, 2013). This spurred the researchers to further study the association between the comprehensive inflammation score and cancer prognosis.

In addition to blood parameters, some inflammatory response-related genes have also been used to assess distant metastasis of cancer (Clatot et al., 2014). The C-C motif chemokine ligand 22 (CCL22) and C-C chemokine receptor 7 (CCR7) have been reported to be highly expressed in tongue squamous cell carcinoma (TSCC) and associated with poor prognosis such as lymph node metastasis (Kimura et al., 2021; Qi Wang et al., 2021). Gene expression levels of coagulation factor III (F3) contribute to the stratification and prognosis prediction of prostate cancer patients (Peng et al., 2016). Interleukin 10 receptor subunit alpha (IL10RA) regulates tumor immune responses and is implicated in the pathogenesis of colorectal cancer. Interleukin 2 receptor subunit beta (IL2RB) polymorphism is associated with lung cancer susceptibility and involved in lung cancer progression (Zadka et al., 2018; Jia et al., 2019). Nevertheless, the association between the inflammatory response–related genes and the outcomes of HNSCC needs to be further investigated.

In this study, the mRNA profile and relevant clinical characteristics of patients with HNSCC were assessed through public databases. Univariate and multivariate Cox regression algorithms were employed to screen 14 genes in The Cancer Genome Atlas (TCGA) database, namely, CCL22, CCR7, CD48 molecule (CD48), F3, heparin-binding EGF-like growth factor (HBEGF), interleukin 10 (IL10), IL10RA, IL2RB, LCK proto-oncogene, Src family tyrosine kinase (LCK), phosphoinositide-3-kinase regulatory subunit 5 (PIK3R5), presenilin 1 (PSEN1), TIMP metallopeptidase inhibitor 1 (TIMP1), TNF alpha–induced protein 6 (TNFAIP6), and TNF receptor superfamily member 1B (TNFRSF1B). Then, the prognostic characteristics were established, and the Gene Expression Omnibus (GEO) database was used for validation. We then evaluated the genes’ prognostic prediction efficiency in patients with HNSCC, as well as their relationships to the immune infiltrate types and the chemotherapeutic sensitivity. Finally, we performed a quantitative real-time polymerase chain reaction (qRT-PCR) analysis to preliminarily verify the mRNA expression levels of these genes in HNSCC.

## Methods

### Data Collection

Clinical information related to HNSCC and the RNA sequencing data of gene expression were obtained from TCGA (https://portal.gdc.cancer.gov/). The GSE41613 chip was acquired from the GEO (http://www.ncbi.nlm.nih.gov/geo/). Through the Molecular Signatures database (MSigDB), we obtained 200 inflammatory response-related genes. The clinicopathological data, including survival information, age, sex, grade, and clinical stage, were collected. All data used in our articles are publicly available in public databases, and patients without survival information were excluded from further analysis. This work is in accordance with the Helsinki Declaration.

### Building and Validating a Prognostic Inflammatory Response–Related Gene Signature

The Akaike information criterion (AIC) (Akaike, 1974) was founded and developed by Japanese statistician Hirotugu Akaike and is a standard to measure the goodness of fit of statistical models. The smaller the AIC value, the better the model. By univariate Cox analysis, we preliminarily identified the inflammatory response–related genes correlated with prognosis in TCGA database. Subsequently, the significant genes obtained from univariate analysis were applied to multivariable Cox algorithms, and the prognostic feature model was established based on the minimum AIC. The signature risk score was generated in light of each gene’s expression level and its relevant coefficient. The detailed equation was: risk scores = (gene-1 expression × corresponding coefficient) + (gene-2 expression × corresponding coefficient) + … + gene-n expression × corresponding coefficient). The patients were then assigned to high- and low-risk groups based on their median risk score, and the Kaplan–Meier (K-M) curve was utilized to examine the OS differences between the two groups. To evaluate the predictive efficacy of the prognostic risk scoring model, the time-dependent receiver operating characteristics (ROC) curve was drawn, and the area under the curve (AUC) was calculated. Additionally, we compared the AUC of Wu et al. (2021) and Jiang et al. (2021) models. A univariate analysis and a multivariate analysis were performed to explore the independent prognostic significance of age, sex, grade, clinical stage, and risk score. After the above process, the best potential prognostic factors were selected and converted into visual nomograms of 1-, 3-, and 5-year OS. To estimate the nomogram’s clinical utility, decision curve analysis (DCA) was utilized. Finally, the above genes and prognostic signatures were tested and verified in the GEO data set.

### Investigation of Tumor Immune Infiltration

To comprehend the relevance among prognostic risk models and immune cell infiltration levels, we performed seven noncontroversial methods for calculating the immune infiltration status of HNSCC patients, including XCELL (Aran, 2020), TIMER (Li et al., 2020), QUANTISEQ (Plattner et al., 2020), MCPCOUNTER (Dienstmann et al., 2019), EPIC (Racle et al., 2017), CIBERSORT-ABS (Tamminga et al., 2020), and CIBERSORT (Chen et al., 2018). The obtained correlation coefficients were plotted as lollipop diagrams. Furthermore, to study the correlations between immune checkpoint expression levels and prognostic risk scores, the level of immune checkpoint genes among the two groups was compared by the Wilcoxon test.

### Chemotherapy Sensitivity Analysis

The half inhibitory concentration (IC50) represents the level of drug required to inhibit tumor cells by 50%. The lower the IC50, the more sensitive the drug. In the TCGA cohort, to assess whether this risk model can guide chemotherapy in patients with HNSCC, we calculated the IC50s of commonly used chemotherapeutics with the “pRRophetic” package. The American Joint Committee on Cancer (AJCC) guidelines suggest anticancer drugs, such as cisplatin, docetaxel, lapatinib, methotrexate, and rapamycin, for use in the treatment of HNSCC. The Wilcoxon measure was used to analyze the discrepancies of IC50s among the high- and low-risk groups, and the outcomes were plotted as a box chart.

### Verification by Quantitative Real-Time Polymerase Chain Reaction

Ten pairs of HNSCC tissues and adjacent noncancerous tissues from the First Affiliated Hospital of Anhui Medical University, approved by the ethics committee, were used in this study. Each participant filled out an informed consent form. The samples were from HNSCC patients who had undergone surgical resection but had not previously received chemotherapy or radiation. The total RNA was extracted from the tissue using TRIzol technology and stored in liquid nitrogen until needed. The Revert Aid First Strand cDNA Synthesis Kit (Thermo Fisher Scientific) was used to reverse transcribe the extracted RNA into cDNA for further analysis, while the concentration of the cDNA was measured to ensure that it meets the standard of use. Supplementary Table S1 shows the primer sequences for PCR. As an internal control, GAPDH was used, and the samples were recorded. Transcriptome levels were collected and differences were compared using the Wilcoxon test.

### Statistical Analysis

The prognostic genes were selected by univariate Cox regression analysis. The K-M method was performed to analyze the distinctions of the OS between high- and low-risk groups, and the statistical significance was obtained by log-rank test. Multivariate Cox regression analyses were used to identify the independent risk factors for the outcomes. The prognostic performance of risk scores on survival prediction was evaluated by ROC curve, and the AUC value was calculated. Spearman correlation analysis was used to test the relevance among the risk score of prognostic models and the immune cell levels. IC50 between the two groups was compared by the Wilcoxon test. The differences in gene expressions between the HNSCC tissues and adjacent non-cancer tissues were tested by Wilcoxon test. All graphics and statistical analyses were completed in R (version 4.1.0) and GraphPad Prism (version 9.0.0).

## Results

### Distinguishing Prognostic Inflammatory Response–Related Genes in Head and Neck Squamous Cell Carcinoma

**FIGURE 1:**
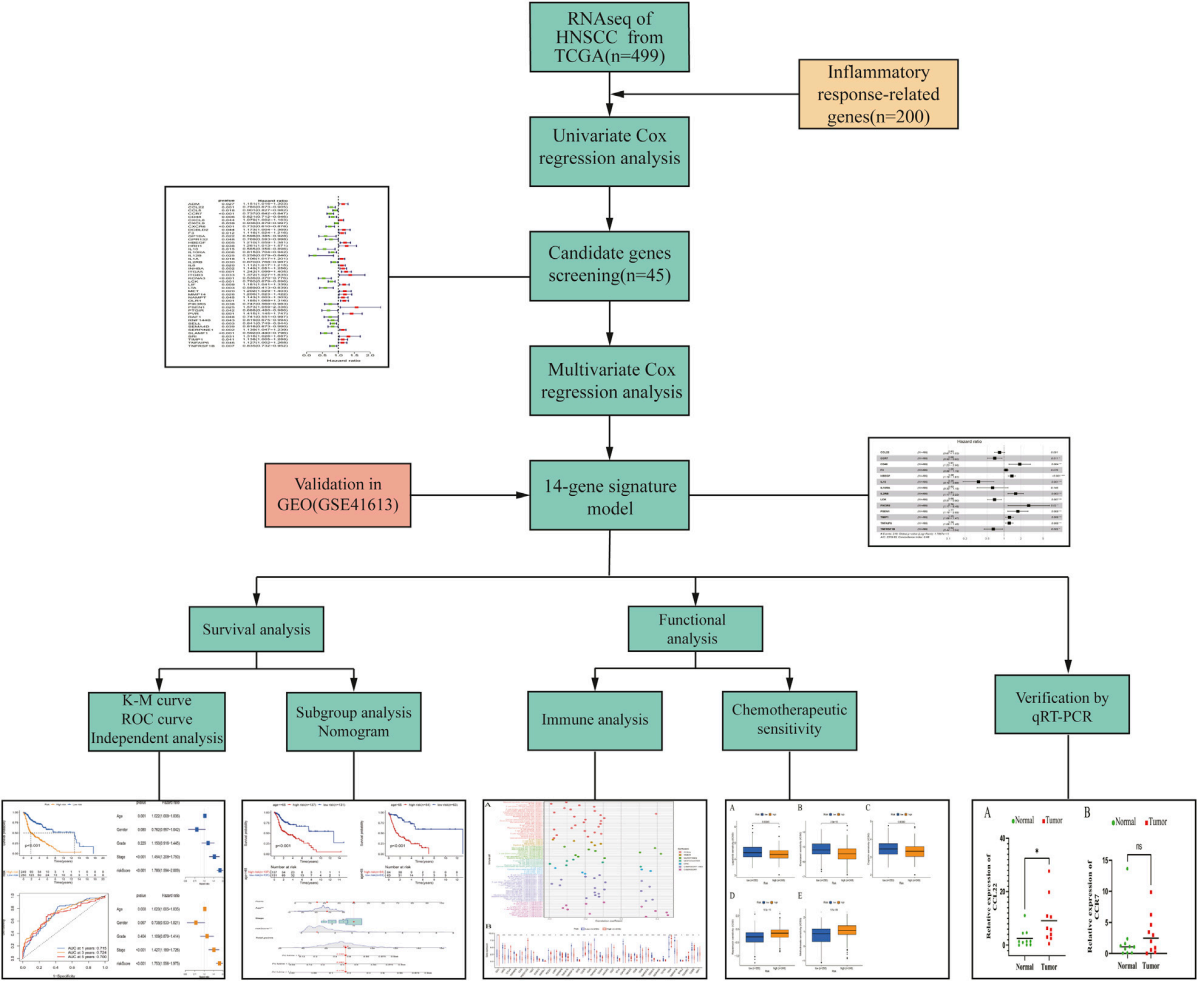
Flow chart of the study.

We summarized the detailed flow chart of Figure 1. The study population included TCGA and GSE41613 data sets. To investigate the potential roles of prognostic inflammatory response-related genes in HNSCC, a total of 200 inflammatory response–related genes were obtained through the MSigDB (Supplementary Table S2). The univariate Cox proportional hazards regression method suggested that 45 of these genes were related to OS and were preserved as prognostic indicators. Twenty-two genes revealed positive coefficients that signified that a higher expression of these genes was accompanied by shorter survival, and another 23 genes had negative coefficients, which showed that high levels of these genes were associated with longer OS (Table 1).

**TABLE 1:** The results of the association between 45 inflammatory response–related genes expression and OS from univariate cox regression analysis.

Gene-ID	HR	95%CI	*p*-value
ADM	1.151	1.016–1.303	0.027
CCL22	0.780	0.673–0.905	0.001
CCL5	0.901	0.827–0.982	0.018
CCR7	0.737	0.642–0.847	0.000
CD48	0.821	0.712–0.946	0.006
CXCL8	1.079	1.002–1.163	0.044
CXCL9	0.936	0.879–0.997	0.039
CXCR6	0.732	0.610–0.878	0.001
DCBLD2	1.173	1.004–1.369	0.044
F3	1.116	1.024–1.216	0.012
GP1BA	0.598	0.385–0.928	0.022
GPR132	0.769	0.593–0.998	0.048
HBEGF	1.210	1.059–1.381	0.005
HRH1	1.261	1.013–1.571	0.038
IL10	0.565	0.356–0.896	0.015
IL10RA	0.815	0.704–0.942	0.006
IL12B	0.258	0.079–0.846	0.025
IL1A	1.106	1.017–1.201	0.018
IL2RB	0.870	0.768–0.987	0.030
IL6	1.112	1.017–1.215	0.020
INHBA	1.149	1.051–1.256	0.002
ITGA5	1.242	1.099–1.405	0.001
ITGB3	1.372	1.027–1.835	0.033
KCNA3	0.536	0.370–0.776	0.001
LCK	0.780	0.679–0.896	0.000
LIF	1.181	1.041–1.339	0.009
LTA	0.589	0.413–0.839	0.003
MET	1.202	1.029–1.403	0.020
MMP14	1.206	1.023–1.422	0.026
NAMPT	1.143	1.003–1.303	0.045
OLR1	1.185	1.068–1.316	0.001
PIK3R5	0.747	0.568–0.983	0.038
PSEN1	1.573	1.059–2.336	0.025
PTGIR	0.688	0.480–0.986	0.042
PVR	1.415	1.145–1.747	0.001
RAF1	0.741	0.551–0.997	0.048
RNF144B	0.819	0.675–0.994	0.043
SELL	0.841	0.749–0.944	0.003
SEMA4D	0.816	0.673–0.990	0.039
SERPINE1	1.139	1.047–1.239	0.002
SLAMF1	0.592	0.440–0.796	0.001
SRI	1.316	1.026–1.687	0.031
TIMP1	1.138	1.005–1.288	0.041
TNFAIP6	1.127	1.002–1.268	0.046
TNFRSF1B	0.835	0.732–0.952	0.007

### Establishment and Verification of Prognostic Inflammatory Response–Related Signature

**FIGURE 2:**
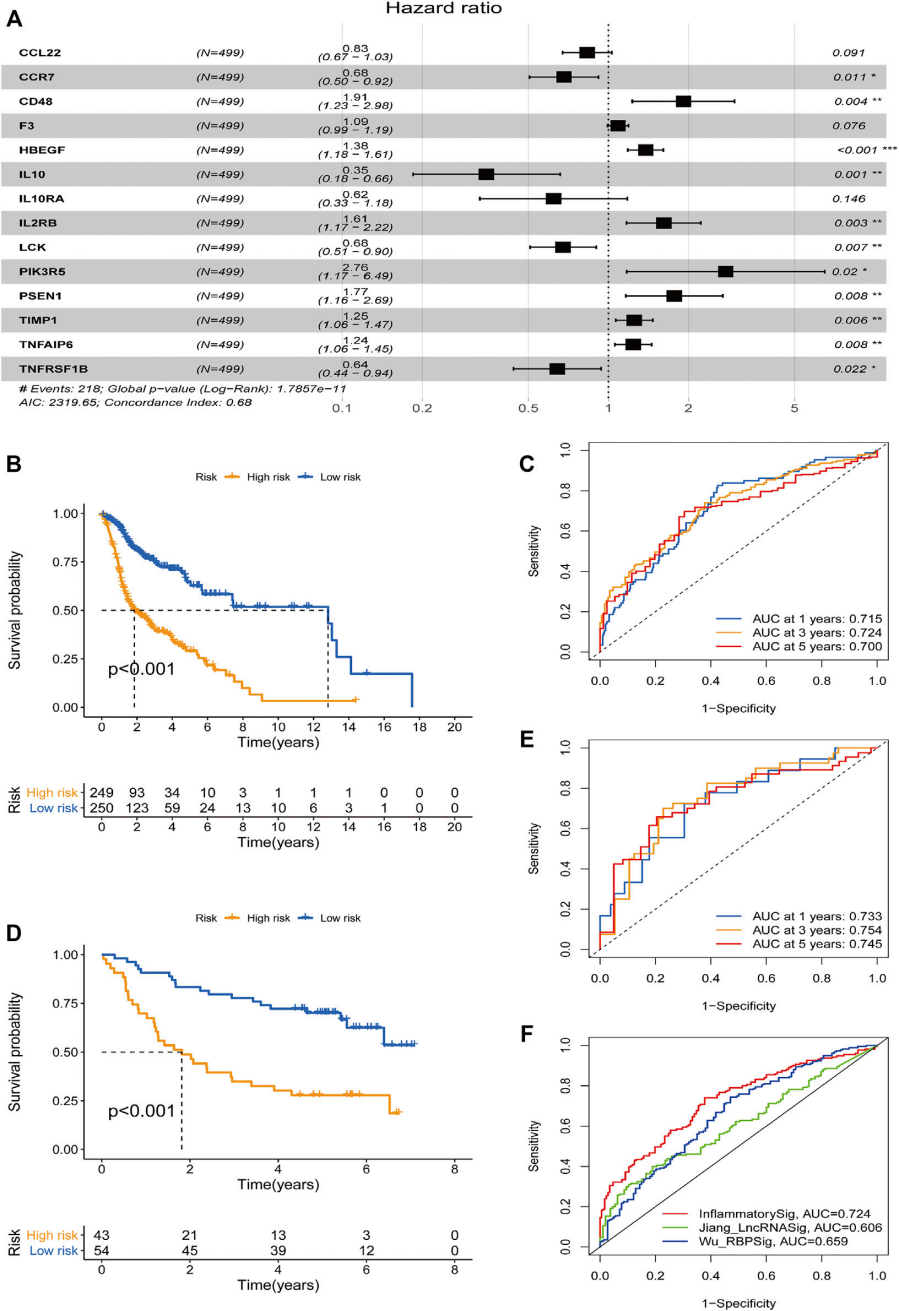
Construction and prognostic analysis of the 14-gene signature model. **(A)** Forest plot showing 14 prognostic inflammatory response-related genes in head and neck squamous cell carcinoma based on a minimum Akaike information criterion (2319.65) value multivariate Cox results. *p < 0.05, **p < 0.01, ***p < 0.001. HR < 1 means protective factors, HR > 1 means risk factors. Kaplan–Meier curve analysis of the inflammatory response–related gene signature in The Cancer Genome Atlas (TCGA) data set **(B)** and the Gene Expression Omnibus (GEO) data set **(D)**. Receiver operating characteristics (ROC) curve analysis of the inflammatory response–related gene signature of 1-, 3-, and 5-year in the TCGA data set **(C)** and GEO data set **(E)**. **(F)** A comparison of the 3-year ROC curve with Wu’s and Jiang’s models.

The relevant data of the above 45 genes were further processed by multivariate analysis. Fourteen genes with a minimum AIC value of 2319.65 were selected and estimated by the R software to develop a prognostic risk model (Figure 2A). The corresponding coefficients are shown in Supplementary Table S3. CD48, F3, HBEGF, IL2RB, PIK3R5, PSEN1, TIMP1, and TNFAIP6 were considered as risk factors with hazard ratio (HR) values greater than 1, whereas the remaining six genes (CCL22, CCR7, IL10, IL10RA, LCK, and TNFRSF1B) were considered protective factors, with HR values less than 1. The risk score was measured in the light of the expression values of the genes as follows: score = (−0.184 × CCL22 expression level) + (−0.386 × CCR7 expression level) + (0.649 × CD48 expression level) + (0.083 × F3 expression level) + (0.322 × HBEGF expression level) + (−1.055 × IL10 expression level) + (−0.474 × IL10RA expression level) + (0.479 × IL2RB expression level) + (−0.392 × LCK expression level) + (1.014 × PIK3R5 expression level) + (0.570 × PSEN1 expression level) + (0.223 × TIMP1 expression level) + (0.215 × TNFAIP6 expression level) + (−0.442 × TNFRSF1B expression level). Using the median risk score as the cutoff point, all patients in the TCGA group (*n* = 499) were separated into a high-risk group (*n* = 249) and a low-risk group (*n* = 250). The survival curve illustrated that the patients with low risk had an obviously longer OS than those in the high-risk group (*p* < 0.001; Figure 2B). To evaluate the model’s performance, the ROC curve was generated, and the AUC was 0.715 at 1 year, 0.724 at 3 years, and 0.700 at 5 years (Figure 2C). To further test the stability of the signature, we evaluated it in the GEO cohort. Consistent with the conclusions of the TCGA cohort, the patients with low risk tended to have a longer survival time (Figure 2D). Furthermore, a similar result was obtained in the ROC curves, and the AUCs at 1, 3, and 5 years of the inflammatory response–related signature were 0.733, 0.754, and 0.745, respectively (Figure 2E). In addition, to verify the optimality of the risk model, we not only drew the 1-, 3-, and 5-year ROC curves but also compared the 3-year ROC curve with Wu et al. (2021) and Jiang et al. (2021) models (Figure 2F). The results demonstrated that the inflammatory response–related signature had better performance.

### Association Between Prognostic Models and Clinicopathological Characteristics

**FIGURE 3:**
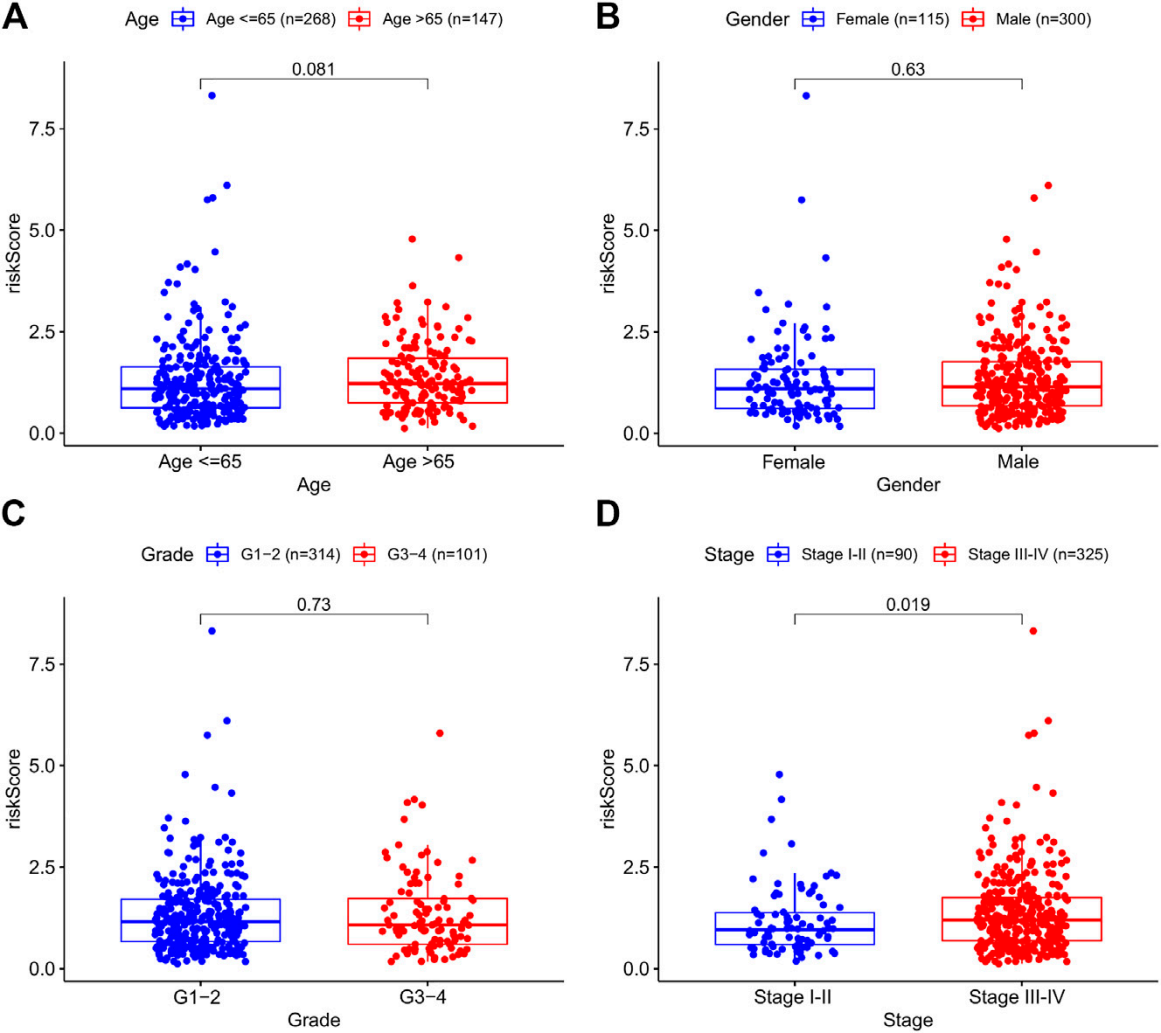
The correlations between the risk model and clinical factors. **(A)** Age. **(B)** Gender. **(C)** Grade. **(D)** Stage.

To investigate the relationship between the prognostic model and clinical features, we first examined the distribution of risk values among clinical features. As shown in Figures 3A–C, risk scores did not differ substantially when age, sex, and grade were considered. By contrast, stages III–IV had a much higher risk score than stages I–II (*p* = 0.019) (Figure 3D). These findings suggested that higher risk scores were associated with higher malignancy in HNSCC.

**FIGURE 4:**
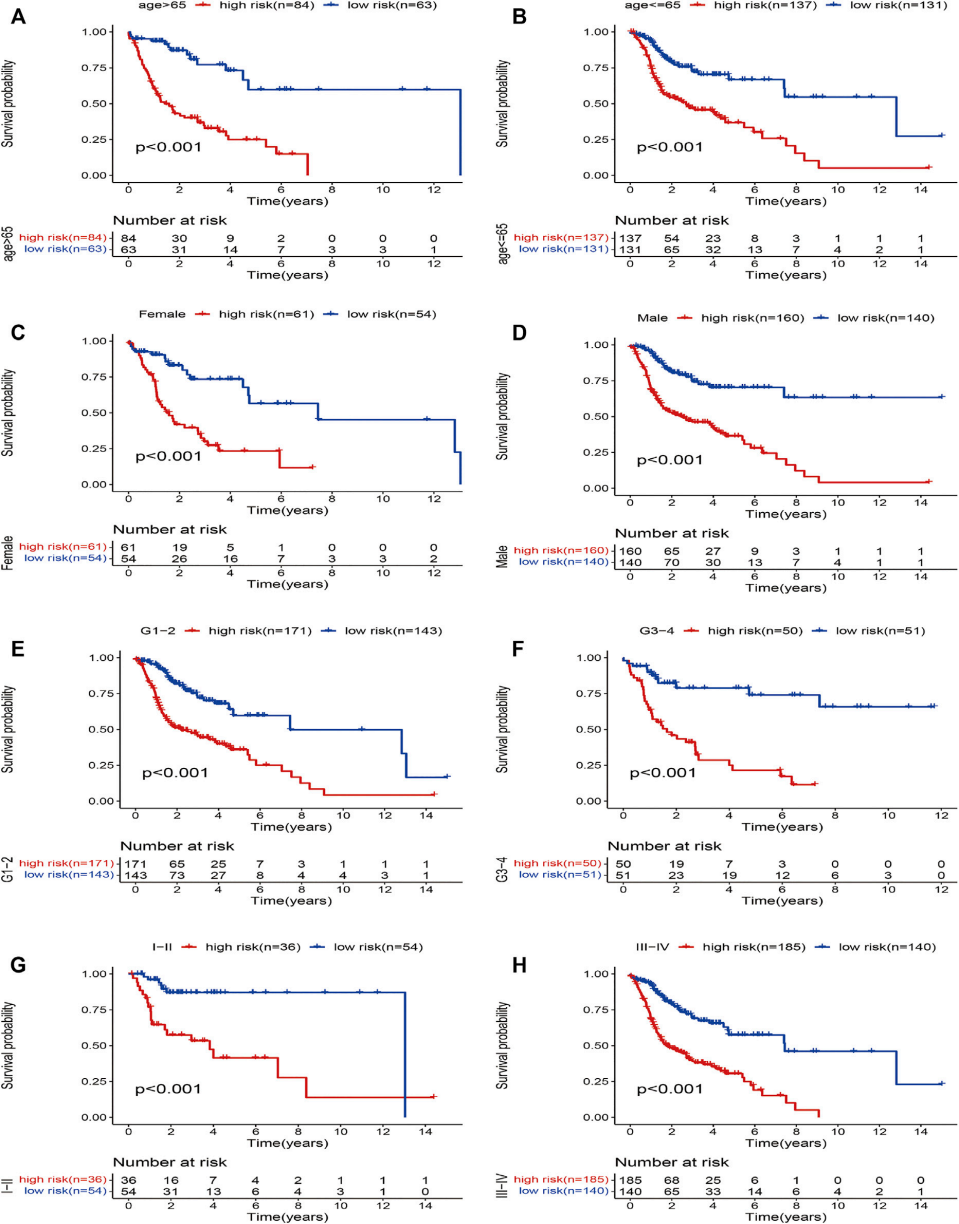
Kaplan–Meier survival curves for the high- and low-risk groups stratified by clinical factors. **(A)** age *>*65 years, **(B)** age ≤65 years, **(C)** gender (female), **(D)** gender (male), **(E)** grades 1–2, **(F)** grades 3–4, **(G)** stages I–II, and **(H)** stages III–IV.

Furthermore, to investigate the prognostic value of the model in HNSCC patients after stratification according to clinicopathological variables, we divided the patients into subsets based on their age, gender, grade, and stage to plot the K-M survival curve. The samples were classified into eight subgroups: younger (≤65 years) and older (>65 years), male and female, earlier grade and advanced grade, and earlier stage and advanced stage. We chose the previous cutoff value, and patients in each subgroup were further assigned to high- and low-risk groups. For all the different subgroups, those in the high-risk group had an obviously shorter OS than their low-risk counterparts (Figures 4A–H). The results suggested that the risk model can anticipate the outcomes of patients with HNSCC without considering clinicopathological subgroups. In sum, the risk signature exerts critical roles in determining the prognosis of patients with HNSCC.

### Independence of the Prognostic Model

**FIGURE 5:**
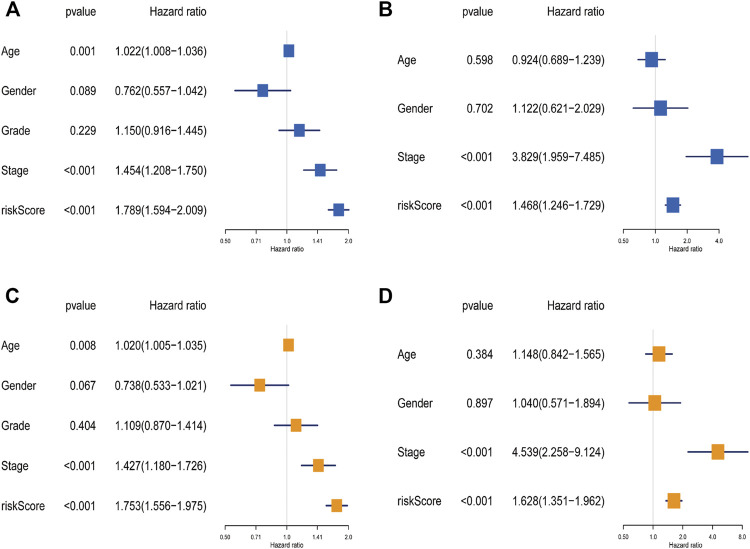
The independence identification of the risk model. Univariate cox regression analysis and multivariate cox regression analysis were performed in The Cancer Genome Atlas data set **(A,C)** and the Gene Expression Omnibus data set **(B,D)**.

To clarify whether the signature was an independent prognostic variable for OS, the risk score and other clinical features (age, gender, grade, and clinical stage) were analyzed. Univariate analysis suggested that the risk score was greatly related to OS (TCGA data set: HR = 1.789, 95% CI = 1.594–2.009, *p* < 0.001; GEO data set: HR = 1.468, 95% CI = 1.246–1.729, *p* < 0.001; Figures 5A,B). Further multivariate Cox analysis excluded other confounding factors, and the results demonstrated that the risk score was still an independent prognostic tool for the OS in the TCGA data set (HR = 1.753, 95% CI = 1.556–1.975, *p* < 0.001) and the GEO data set (HR = 1.628, 95% CI = 1.351–1.962, *p* < 0.001; Figures 5C,D). In addition, age and clinical stage were also confirmed as independent predictors for OS (Figure 5).

### Nomogram Construction and Verification

**FIGURE 6:**
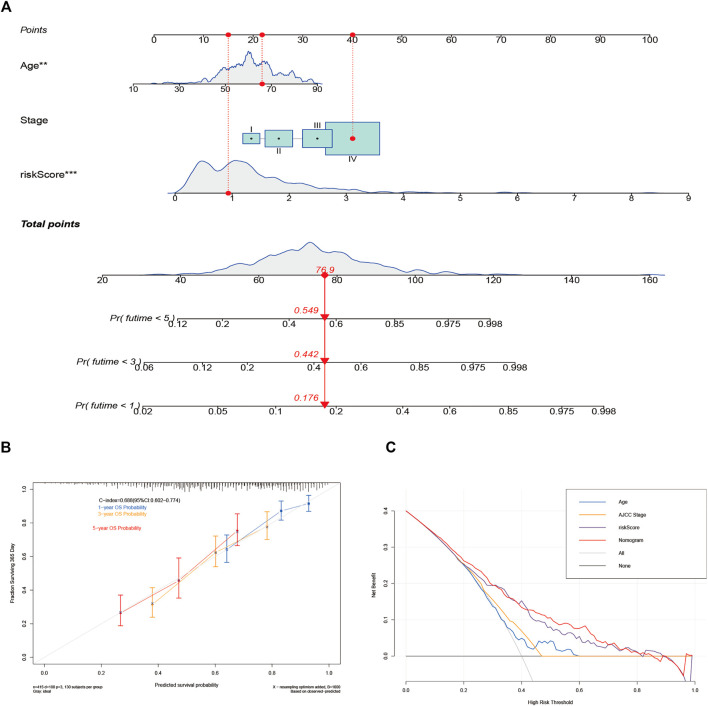
Construction and validation of the nomogram for predicting the overall survival (OS) of patients with head and neck squamous cell carcinoma. **(A)** The nomogram for predicting the OS of patients at 1, 3, and 5 years. **(B)** Calibration curves of the nomogram for OS prediction at 1, 3, and 5 years. **(C)** Decision curve analysis for the prediction of 1-, 3-, and 5-year OS.

A nomogram with an integrated prognostic model and other clinical parameters was developed to predict the OS of HNSCC patients and to assist doctors in providing better therapy (Figure 6A). Then, a calibration curve was created to determine the nomogram’s reliability (Figure 6B). Moreover, DCA was performed to analyze the net benefits of none, all, age, AJCC stage, risk score, and the nomogram to verify the clinical utility of the nomogram. As shown in Figure 6C, the nomogram had a better potential for clinical utility compared with the other five groups. Thus, the risk model provided effective prognosis predictive value and delivered some net benefits that may be useful for patients with HNSCC.

### Association of Risk Score With Immune Infiltrating Cells

**FIGURE 7:**
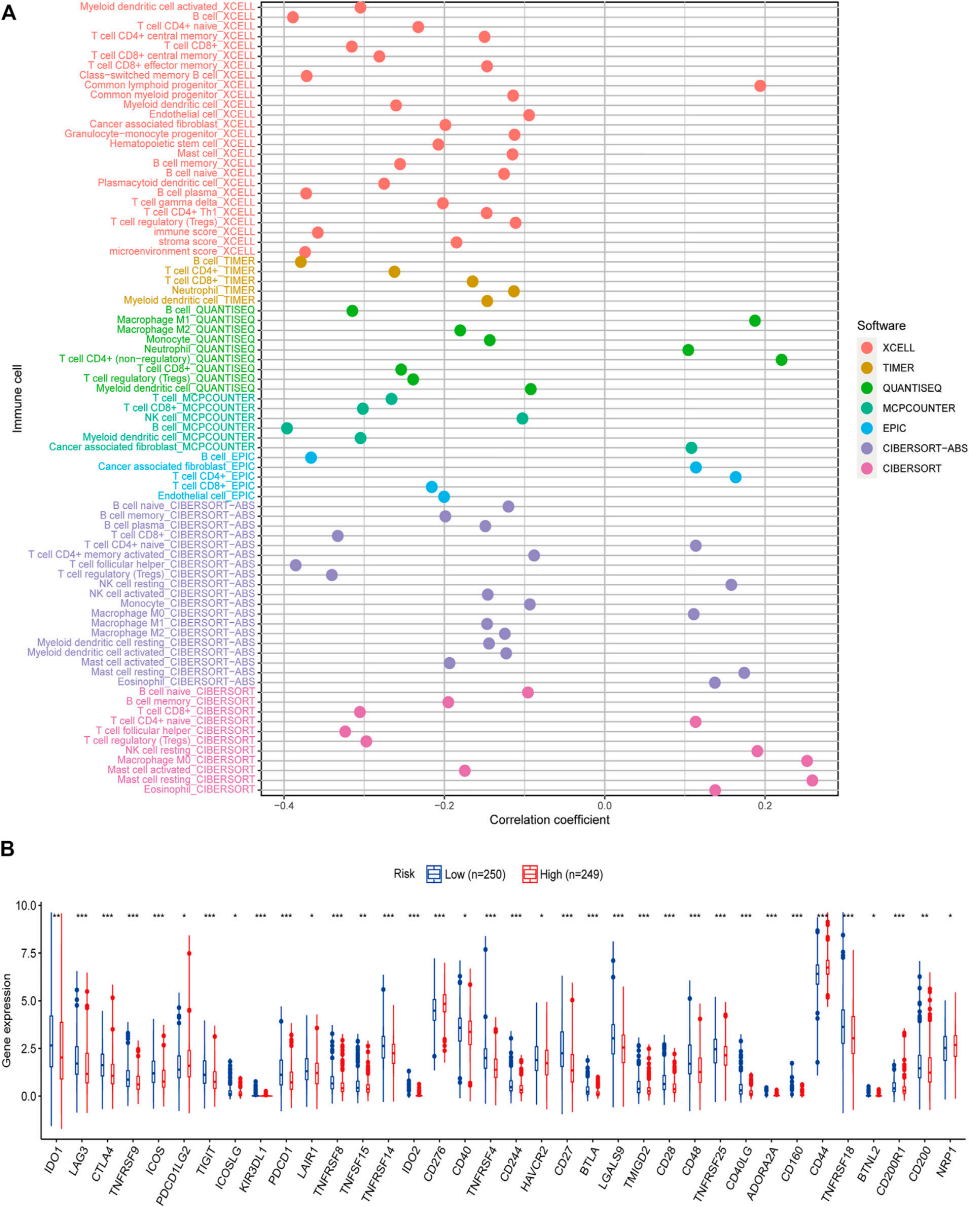
Correlation of tumor-infiltrating cells and immune checkpoints with risk scores. **(A)** A lollipop diagram shows that high-risk score was positively related to M0, M1 macrophages, and resting NK cells infiltrating, whereas it was negatively associated with B cells, CD8 T cells, M2 macrophages, myeloid dendritic cells, and monocytes infiltrating in head and neck squamous cell carcinoma patients. **(B)** Expression of immune checkpoints among high- (*n* = 249) and low-risk groups (*n* = 250). *p < 0.05, **p < 0.01, ***p < 0.001.

To investigate the efficiency of inflammatory response–related genes on the status of the tumor microenvironment, seven algorithms were applied to investigate the correlations between the level of immune cells and the model-predicted risk score. After Spearman correlation analysis was conducted, we found that the risk score was positively linked to M0, M1 macrophages, and resting NK cells infiltrating, while being negatively associated with B cells, CD8 T cells, M2 macrophages, myeloid dendritic cells, and monocytes infiltrating in the HNSCC samples (Figure 7A; Supplementary Table S4). We then detected the expression levels of immune checkpoints among samples with different risk scores, and differences were further found in the expressions of some immune checkpoint genes between the two groups (Figure 7B). The outcomes revealed that these genes might be prospective indicators for the regulation of immune activity in the immune microenvironment and may play an important role in checkpoint inhibitor-based immunotherapies.

### Correlation Between the Prognostic Signature and Chemosensitivity

**FIGURE 8:**
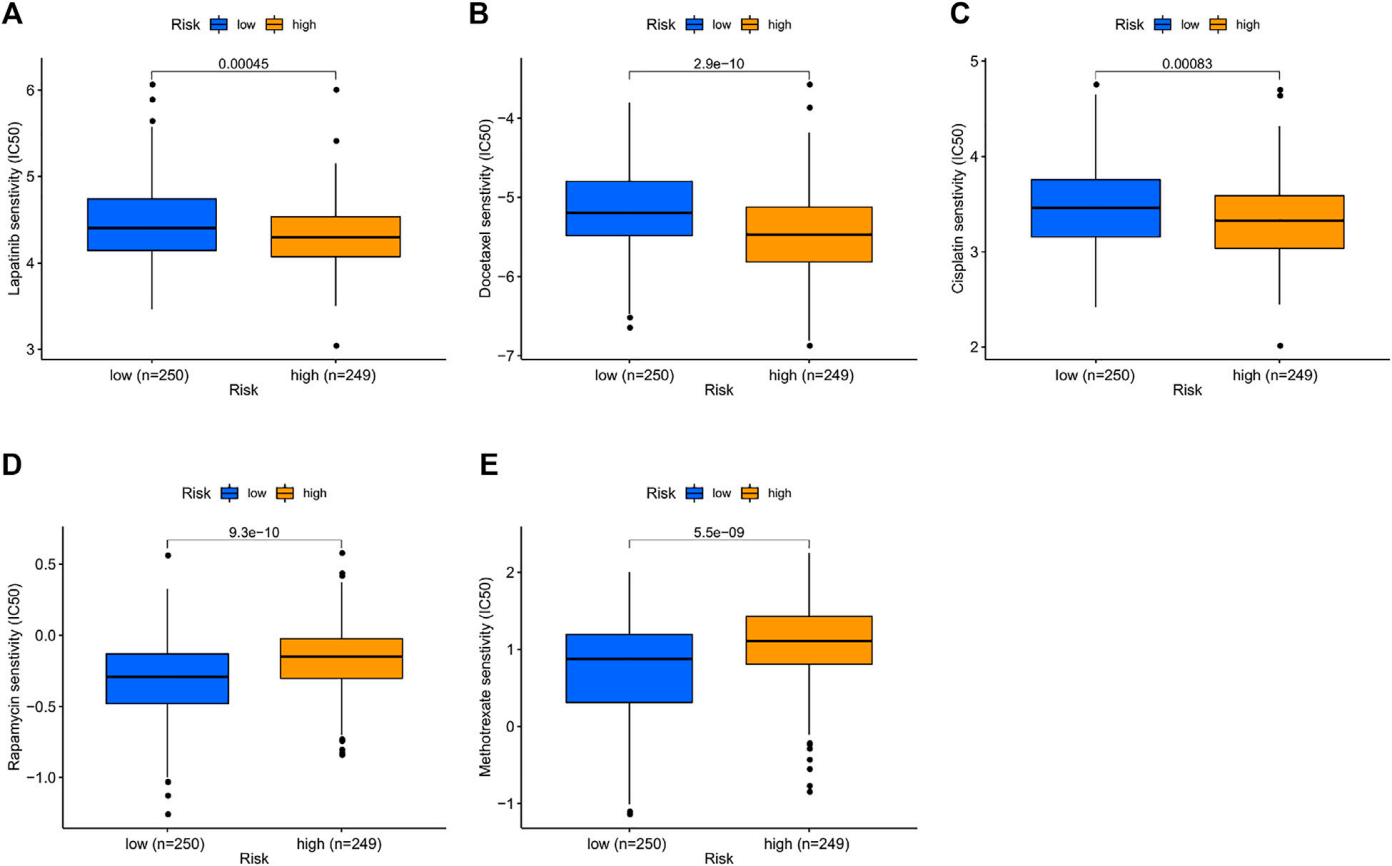
Prediction sensitivity of chemotherapy benefit in high- (*n* = 249) and low-risk groups (*n* = 250). **(A)** Lapatinib. **(B)** Docetaxel. **(C)** Cisplatin. **(D)** Rapamycin. **(E)** Methotrexate.

Chemotherapy is an important part of the comprehensive treatment of HNSCC. Therefore, we sought to determine whether there is a correlation between risk scores and patient sensitivity to commonly used chemotherapeutic agents. The results showed that drugs such as lapatinib (*p* < 0.001; Figure 8A), docetaxel (*p* < 0.001; Figure 8B), and cisplatin (*p* < 0.001; Figure 8C) had lower IC50s in the high-risk group, while rapamycin (*p* < 0.001; Figure 8D) and methotrexate (*p* < 0.001; Figure 8E) were lower in the low-risk group. In short, high-risk groups were more sensitive to lapatinib, docetaxel, and cisplatin, while low-risk groups responded better to rapamycin and methotrexate. Therefore, this model shows great potential for predicting chemosensitivity and may help clinicians choose the best chemotherapy regimen.

### Expression Levels of C-C Motif Chemokine Ligand 22 and Interleukin 10 was Upregulated in Quantitative Real-Time Polymerase Chain Reaction

**FIGURE 9:**
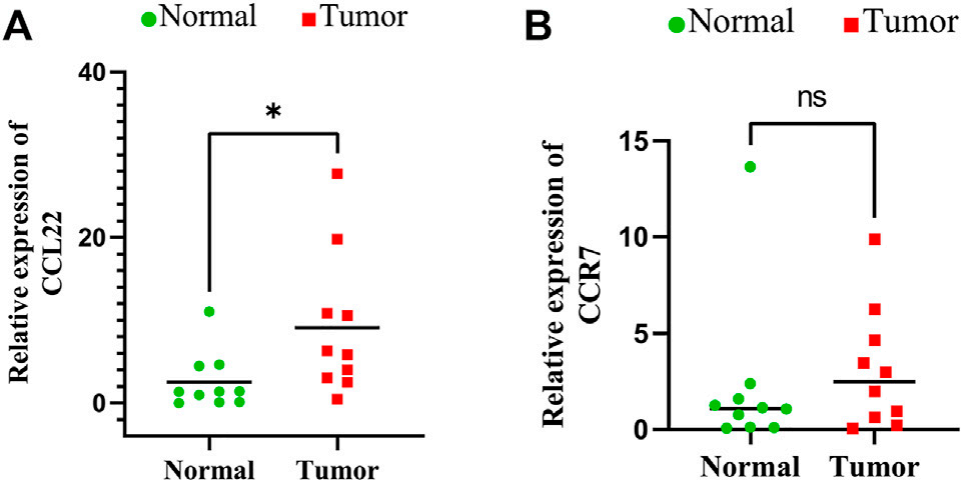
Expressions of signature genes CCL22 and IL10 were upregulated in head and neck squamous cell carcinoma. **(A)** qPCR analysis of CCL22 mRNA levels in tumor and adjacent normal tissues. **(B)** qPCR analysis of IL10 mRNA levels in tumor and adjacent normal tissues.

qRT-PCR was used to examine the relative mRNA expression levels of these genes in 10 pairs of HNSCC and adjacent non-cancer tissues. As shown in Figure 9, the expression of CCL22 and IL10 was much higher in HNSCC tissues than in adjacent tissues. However, no significant differences were observed in the mRNA expression levels of the remaining 12 genes between HNSCC tissues and normal tissues (Supplementary Figure S1). This may be related to the small number of samples we tested.

## Discussion

With the progress of medical technology, various treatments for HNSCC have been fully developed. However, due to the lack of reliable biomarkers, we are often unable to effectively predict the therapeutic efficacy of HNSCC treatments. A growing number of researchers have shown that inflammation response plays a significant role in the pathophysiology and progression of HNSCC and is associated with patient outcomes (Rassouli et al., 2015; Zhou et al., 2020). Serum indicators linked with inflammation, such as the neutrophil/lymphocyte ratio, platelet/lymphocyte ratio, and lymphocyte/monocyte ratio, have been shown to be effective at forecasting the prognosis of HNSCC in recent years (Zhou et al., 2019; Valdes et al., 2020). However, inflammatory response-related genes, as prognostic predictors for HNSCC, have not been developed. According to the existing literature, an RNA-binding protein-based signature (Wu et al., 2021) and a three-lncRNA signature (Jiang et al., 2021) predicted the 3-year OS for HNSCC with AUCs of 0.659 and 0.606, respectively. The inflammatory response-related gene signature built in the present study shows better validity than the above gene signatures. Moreover, another important advantage of our research is that we evaluated the value of the risk models in predicting immune cell infiltration and chemotherapy sensitivity, further increasing the model’s clinical utility and providing potential biomarkers for clinical therapeutics.

In our research, we employed RNA expression data and 200 inflammatory response-related genes to systematically analyze OS in patients with HNSCC. In the TCGA data set, we built a prognostic signature containing 14 inflammatory response-related genes to establish risk stratification and predict clinical outcomes. Patients with HNSCC were compartmentalized into low-risk and high-risk groups using the median risk score. We found that the low-risk group was significantly correlated to a longer OS, which was effectively validated in the GEO database. Further analysis revealed that the correlation between the risk models and OS in HNSCC patients was independent of clinicopathological subgroups. In addition, independent analysis showed that the prognostic risk model was an independent prognostic factor for HNSCC. Then, by combining the inflammatory prognostic signature with other prognostic variables, we developed a simple-to-use prediction nomogram model, which was helpful in predicting 1-, 3-, and 5-year OS and may prove beneficial in the clinical treatment of HNSCC patients.

The inflammatory response–related gene signature consisted of 14 genes (CCL22, CCR7, CD48, F3, HBEGF, IL10, IL10RA, IL2RB, LCK, PIK3R5, PSEN1, TIMP1, TNFAIP6, and TNFRSF1B). Previous research has shown that these 14 genes play a role in cancer progression. CCL22 is one of the several Cys-Cys (CC) cytokine genes, mainly produced by M2-like tumor-associated macrophages in the tumor microenvironment. CCL22 plays an important role in regulating regulatory T-cell migration by binding to its receptor CCR4 (Rohrle et al., 2020). Studies have found that high expression of CCL22 in the tumor microenvironment is closely related to lymph node recurrence in TSCC and poor prognosis of cervical cancer (Kimura et al., 2021; Wang et al., 2019). Our study also confirmed this, as we found that CCL22 mRNA levels were significantly upregulated in HNSCC tissues by qRT-PCR. Found in a variety of lymphoid organs, CCR7 has been shown to activate B and T lymphocytes, regulate the movement of memory T cells to inflamed tissues, and promote dendritic cell growth. Furthermore, CCR7 is required for the growth of lymph nodes and the construction of their follicular functional structures, as well as for the guidance of cells to lymphoid tissue (Fuss et al., 2021). Therefore, like CCL22, CRR7 plays a crucial role in the migration of tumor cells to the lymphatic system and makes a crucial contribution to the metastasis and expansion of TSCC (Qi Wang et al., 2021). As a co-stimulatory and adhesion molecule, CD48 is mainly synthesized by hematopoietic cells, especially antigen-presenting cells. CD48 interacts with CD2 and participates in various innate immune responses, playing a key role in regulating immune activation or suppression (Mcardel et al., 2016). In addition, the expression of CD48 has been shown to be upregulated throughout the development of glioblastoma, and patients with high CD48 expression have a poorer prognosis (Zou et al., 2019). HBEGF can activate growth factor activity and heparin-binding activity, which is involved in multiple processes, such as promoting wound healing, and plays a role in the epidermal growth factor receptor pathway (Higashiyama et al., 1991). HBEGF is abundantly produced in ovarian and breast cancers, and its high expression can promote the tumor cell or macrophage paracrine invasion loop, thereby promoting tumor infiltration, invasion, and metastasis (Zhou et al., 2014). F3, also known as tissue factor (TF), is a protein encoded by a gene in cells responsible for initiating the blood clotting process. It promotes the hypercoagulable state in cancer patients by acting as a high-affinity receptor for coagulation factor VII (Liu et al., 2015). Furthermore, F3 promotes tumorigenesis, angiogenesis, and tumor cell migration and metastasis, all of which contribute to tumor expansion. According to previous studies, F3 gene expression levels were significantly elevated in patients with advanced lung cancer and were associated with poorer survival (Régina et al., 2009). IL10 is an interleukin mainly produced by monocytes, with multiple effects on innate and adaptive immunity and inflammatory processes. It inhibits the production of Th1 cytokines, MHC class II antigens, and co-stimulatory molecules in macrophages and also helps B cells survive, proliferate, and produce antibodies. Importantly, it is involved in the control of tumor cell proliferation and invasion through the JAK/STAT signaling pathway (Béguelin et al., 2015). In this study, the expression level of IL10 was significantly increased in HNSCC tissues, suggesting that IL10 may be a potential prognostic factor in HNSCC. As a receptor for IL10, IL10RA can regulate tumor immune responses and is highly expressed in HNSCC tissues (Juncheng Wang et al., 2021). However, in our study, its expression did not differ between tumors and adjacent tissues, which may be related to our small sample size. IL2RB is one of the receptors of IL2 that is involved in T-cell–mediated immune responses, and its binding to IL2 promotes the growth of NK92 cells and its anticancer function (Jounaidi et al., 2017). LCK is an important factor regulating the motility of oral cancer cells and is associated with the invasion and metastasis of oral cancer cells. LCK inhibitors could be used to regulate glioma cell movement, cancer invasion, and stem cell gene expression (Zepecki et al., 2018). This suggests that LCK may become a new target gene for cancer therapy. PIK3R5 gene has predictive potential for lymph node metastasis in breast cancer, while PSEN1 downregulation promotes the chemoradiotherapy resistance of esophageal carcinoma (Meng et al., 2016; Paula et al., 2017). TIMP1 and TNFRSF1B genes have been confirmed to take part in the pathogenesis of colorectal cancer and have prognostic value (Yu et al., 2014; Huang et al., 2019). Downregulation of TNFAIP6 can inhibit the proliferation, invasion, and metastasis of gastric cancer (GC). Previous research demonstrated that increased TNFAIP6 expression is linked to the level of infiltration, lymph node metastases, and poor outcome in GC patients, implying that TNFAIP6 may be a workable therapeutic target for GC (Zhang et al., 2021). Our findings demonstrate these genes can be applied to forecast HNSCC outcomes, although their mechanisms need to be further explored.

It has been increasingly recognized that the tumor microenvironment is closely correlated with the occurrence and progression of cancers. The results of existing studies have shown that immune component imbalance is a significant factor causing the short survival of patients with cancer (Li et al., 2017). To further understand the association among risk scores and tumor infiltrating immune cells, we performed seven established algorithms for immune infiltration analysis. Due to the heterogeneity of these methods, internal comparisons were rarely utilized. Through integration analysis, our study showed that the abundance of B cells, CD8 T cells, M2 macrophages, myeloid dendritic cells, and monocytes in the low-risk group was higher, and the high-risk group had higher M0, M1 macrophages, and resting NK cells. Yang et al. (2019) reported that in cervical cancer patients, CD8 T-cell infiltration tended to have a good prognosis, whereas Ali et al. (2016) suggested that increased levels of M0 macrophages led to a poor prognosis in breast cancer. The results of these studies are consistent with ours.

Checkpoint inhibitor immunotherapies significantly improved the prognosis of patients with advanced cancers (Hellmann et al., 2018). We found a meaningful distinction in the expression of immune checkpoints among the two groups in the present study, which suggested that the sensitivity for immunotherapy may also be different. Additionally, our study demonstrated that the high-risk patients were susceptible to chemotherapy drugs, for example, cisplatin, docetaxel, and lapatinib. Therefore, the results of our study may help foresee the immune checkpoint levels and potentially guide immunotherapy and chemotherapeutic decisions. Finally, we carried out qRT-PCR analysis and concluded that mRNA expression levels of CCL22 and IL10 were higher in HNSCC cancer tissues than in normal tissues.

However, our research has some limitations, which does not affect the value of this study. First, we presented bioinformatic evidence by constructing and evaluating 14 inflammatory response-related gene signatures based on traditional statistical methods that can accurately predict the prognosis of HNSCC. Although many reports have proved the practicability of these methods, it is still necessary to develop higher-level statistical techniques to improve the accuracy and stability of the model. Additionally, our outcomes were only verified using the GEO data sets and qRT-PCR, and further validation in additional clinical cases is required. Second, we only analyzed the correlation between inflammatory genes and clinical prognosis, and further experimental studies are required to explore the specific mechanism of these genes in relation to the prognosis of HNSCC. Finally, tobacco, alcohol, HPV, and EBV infections, which are important risk factors for HNSCC, were not included in the analysis. We will continue to investigate this field in the future.

## Conclusion

In summary, we identified 14 prognostic inflammatory response-related genes in HNSCC and developed a new prognostic signature model that was confirmed to be independently related to OS. Furthermore, this signature revealed the immune cell infiltration patterns and chemotherapeutic sensitivity in HNSCC, which can assist in predicting the prognosis and guiding the treatment.

## Data Availability

The datasets presented in this study can be found in online repositories. The names of the repository/repositories and accession number(s) can be found in the article/Supplementary Material.
