# Supplementary figures and images for: A Novel Inflammatory Response–Related Gene Signature Improves High-Risk Survival Prediction in Patients With Head and Neck Squamous Cell Carcinoma
Source: Front Genet. 2022 Apr 11;13:767166. doi: 10.3389/fgene.2022.767166 (PMC9035793; doi:10.3389/fgene.2022.767166)

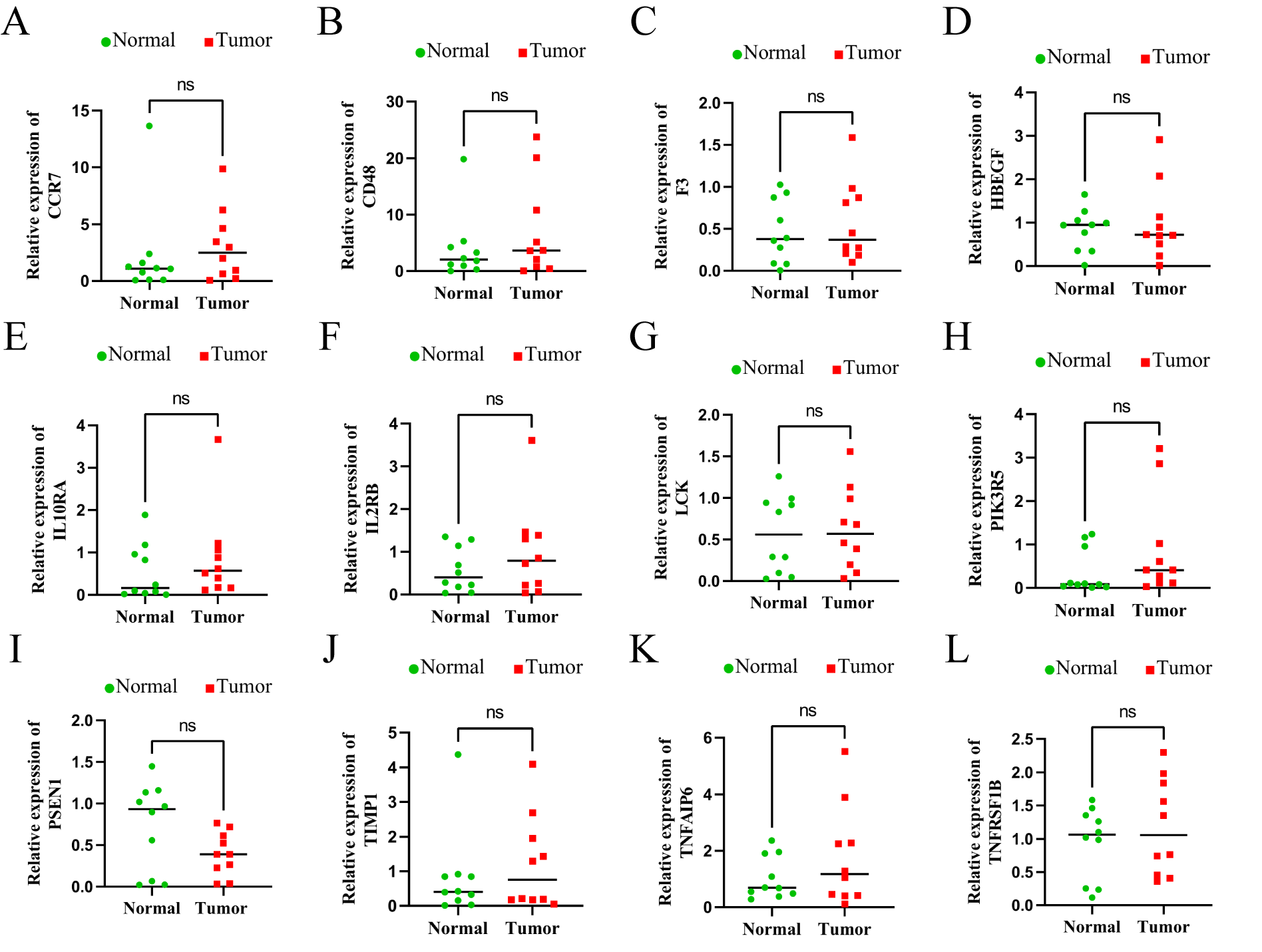

Supplement: Supplementary file 1 [file Image1.TIF]
