# Supplementary material for: A Novel Inflammatory Response–Related Gene Signature Improves High-Risk Survival Prediction in Patients With Head and Neck Squamous Cell Carcinoma
Source: Front Genet. 2022 Apr 11;13:767166. doi: 10.3389/fgene.2022.767166 (PMC9035793; doi:10.3389/fgene.2022.767166)
Supplement: Supplementary file 2 [file DataSheet1.docx]

**Supplemental Information**

**A novel Inflammatory response-related genes signature improves high-risk survival prediction in patients with Head and neck squamous cell carcinoma**

**Yanxun Han, Zhao Ding, Bangjie Chen, Yuchen Liu, Yehai Liu**

**Table S1.** **The primer sequences of qRT-PCR.**

| Primer | Sequence (5’-3’) |
| --- | --- |
| CCL22: F | GAAACACTTCTACTGGACCTCA |
| CCL22: R | TGGCTCAGCTTATTGAGAATCA |
| CCR7: F | CATGCTCCTACTTCTTTGCATC |
| CCR7: R | CACTGTGGCTAGTATCCAGATG |
| CD48: F | AGTCTGACCCTGCCTTTAAATT |
| CD48: R | CTGCCTGAGAACTACAAACAAC |
| F3: F | CAAACCCGTCAATCAAGTCTAC |
| F3: R | GCTTCACATCCTTCACAATCTC |
| HBEGF: F | CAGATCTGGACCTTTTGAGAGT |
| HBEGF: R | TTTCTTCTTTCTTTTCCCGTGC |
| IL10: F | GTTGTTAAAGGAGTCCTTGCTG |
| IL10: R | TTCACAGGGAAGAAATCGATGA |
| IL10RA: F | GTAACAAGGGGATGTGGTCTAA |
| IL10RA: R | AAAGGCAAAGAAGATGATGACG |
| IL2RB: F | CTGAGATCTCGCCACTAGAAG |
| IL2RB: R | GGAAGAAGAAGTAACCCTGGTT |
| LCK: F | GTGTGTGAGAACTGCCATTATC |
| LCK: R | GATTGGAGCCTTCGTAGGTAAC |
| PSEN1: F | CATTATCTAATGGACGACCCCA |
| PSEN1: R | AATGGGGTATAGATTAGCTGCC |
| TIMP1: F | CATCACTACCTGCAGTTTTGTG |
| TIMP1: R | TGGATAAACAGGGAAACACTGT |
| TNFAIP6: F | GTTGCTTGGCTGATTATGTTGA |
| TNFAIP6: R | CTCATCTCCACAGTATCTTCCC |
| TNFRSF1B: F | CGGCTCAGAGAATACTATAGACC |
| TNFRSF1B: R | ACAGAAGACTTTTGCATGTTGG |
| PIK3R5: F | CTTCCACGCTACGTGTTGTG |
| PIK3R5: R | TGAAGTTTGAAGAACCGTGTGAG |
| GAPDH: F | GTATCGTGGAAGGACTCATGAC |
| GAPDH: R | ACCACCTTCTTGATGTCATCAT |

**Table S2.** **The 200 inflammatory response-related genes (submitted as a separate Excel file).**

**Table S3. The corresponding coefficients of multivariate cox regression analysis.**

| Gene-ID | Coefficients | HR | 95% CI | PValue |
| --- | --- | --- | --- | --- |
| CCL22 | -0.184 | 0.832 | 0.672-1.030 | 0.091 |
| CCR7 | -0.386 | 0.680 | 0.505-0.916 | 0.011 |
| CD48 | 0.649 | 1.913 | 1.229-2.978 | 0.004 |
| F3 | 0.083 | 1.087 | 0.991-1.192 | 0.076 |
| HBEGF | 0.322 | 1.380 | 1.183-1.609 | 0.000 |
| IL10 | -1.055 | 0.348 | 0.184-0.658 | 0.001 |
| IL10RA | -0.474 | 0.623 | 0.329-1.179 | 0.146 |
| IL2RB | 0.479 | 1.614 | 1.171-2.225 | 0.003 |
| LCK | -0.392 | 0.675 | 0.507-0.899 | 0.007 |
| PIK3R5 | 1.014 | 2.757 | 1.172-6.488 | 0.020 |
| PSEN1 | 0.570 | 1.768 | 1.163-2.688 | 0.008 |
| TIMP1 | 0.223 | 1.250 | 1.065-1.467 | 0.006 |
| TNFAIP6 | 0.215 | 1.239 | 1.057-1.453 | 0.008 |
| TNFRSF1B | -0.442 | 0.642 | 0.440-0.937 | 0.022 |

**Table S4. The detail comparison results of correlation ship between tumor infiltrating immune cells and risk sore.**

| Symbol | Type | Correlation | P-Value |
| --- | --- | --- | --- |
| B cell_TIMER | TIMER | -0.3790 | 0.0000 |
| T cell CD4+_TIMER | TIMER | -0.2623 | 0.0000 |
| T cell CD8+_TIMER | TIMER | -0.1648 | 0.0002 |
| Neutrophil_TIMER | TIMER | -0.1132 | 0.0114 |
| Myeloid dendritic cell_TIMER | TIMER | -0.1465 | 0.0010 |
| B cell naive_CIBERSORT | CIBERSORT | -0.0958 | 0.0324 |
| B cell memory_CIBERSORT | CIBERSORT | -0.1951 | 0.0000 |
| T cell CD8+_CIBERSORT | CIBERSORT | -0.3054 | 0.0000 |
| T cell CD4+ naive_CIBERSORT | CIBERSORT | 0.1134 | 0.0112 |
| T cell follicular helper_CIBERSORT | CIBERSORT | -0.3237 | 0.0000 |
| T cell regulatory (Tregs)_CIBERSORT | CIBERSORT | -0.2974 | 0.0000 |
| NK cell resting_CIBERSORT | CIBERSORT | 0.1904 | 0.0000 |
| Macrophage M0_CIBERSORT | CIBERSORT | 0.2524 | 0.0000 |
| Mast cell activated_CIBERSORT | CIBERSORT | -0.1745 | 0.0001 |
| Mast cell resting_CIBERSORT | CIBERSORT | 0.2590 | 0.0000 |
| Eosinophil_CIBERSORT | CIBERSORT | 0.1378 | 0.0020 |
| B cell naive_CIBERSORT-ABS | CIBERSORT-ABS | -0.1200 | 0.0073 |
| B cell memory_CIBERSORT-ABS | CIBERSORT-ABS | -0.1988 | 0.0000 |
| B cell plasma_CIBERSORT-ABS | CIBERSORT-ABS | -0.1489 | 0.0008 |
| T cell CD8+_CIBERSORT-ABS | CIBERSORT-ABS | -0.3330 | 0.0000 |
| T cell CD4+ naive_CIBERSORT-ABS | CIBERSORT-ABS | 0.1137 | 0.0111 |
| T cell CD4+ memory activated_CIBERSORT-ABS | CIBERSORT-ABS | -0.0880 | 0.0494 |
| T cell follicular helper_CIBERSORT-ABS | CIBERSORT-ABS | -0.3854 | 0.0000 |
| T cell regulatory (Tregs)_CIBERSORT-ABS | CIBERSORT-ABS | -0.3404 | 0.0000 |
| NK cell resting_CIBERSORT-ABS | CIBERSORT-ABS | 0.1579 | 0.0004 |
| NK cell activated_CIBERSORT-ABS | CIBERSORT-ABS | -0.1459 | 0.0011 |
| Monocyte_CIBERSORT-ABS | CIBERSORT-ABS | -0.0934 | 0.0370 |
| Macrophage M0_CIBERSORT-ABS | CIBERSORT-ABS | 0.1111 | 0.0131 |
| Macrophage M1_CIBERSORT-ABS | CIBERSORT-ABS | -0.1467 | 0.0010 |
| Macrophage M2_CIBERSORT-ABS | CIBERSORT-ABS | -0.1246 | 0.0053 |
| Myeloid dendritic cell resting_CIBERSORT-ABS | CIBERSORT-ABS | -0.1443 | 0.0012 |
| Myeloid dendritic cell activated_CIBERSORT-ABS | CIBERSORT-ABS | -0.1229 | 0.0060 |
| Mast cell activated_CIBERSORT-ABS | CIBERSORT-ABS | -0.1935 | 0.0000 |
| Mast cell resting_CIBERSORT-ABS | CIBERSORT-ABS | 0.1741 | 0.0001 |
| Eosinophil_CIBERSORT-ABS | CIBERSORT-ABS | 0.1374 | 0.0021 |
| B cell_QUANTISEQ | QUANTISEQ | -0.3148 | 0.0000 |
| Macrophage M1_QUANTISEQ | QUANTISEQ | 0.1874 | 0.0000 |
| Macrophage M2_QUANTISEQ | QUANTISEQ | -0.1802 | 0.0001 |
| Monocyte_QUANTISEQ | QUANTISEQ | -0.1434 | 0.0013 |
| Neutrophil_QUANTISEQ | QUANTISEQ | 0.1043 | 0.0198 |
| T cell CD4+ (non-regulatory)_QUANTISEQ | QUANTISEQ | 0.2206 | 0.0000 |
| T cell CD8+_QUANTISEQ | QUANTISEQ | -0.2539 | 0.0000 |
| T cell regulatory (Tregs)_QUANTISEQ | QUANTISEQ | -0.2389 | 0.0000 |
| Myeloid dendritic cell_QUANTISEQ | QUANTISEQ | -0.0922 | 0.0395 |
| T cell_MCPCOUNTER | MCPCOUNTER | -0.2660 | 0.0000 |
| T cell CD8+_MCPCOUNTER | MCPCOUNTER | -0.3017 | 0.0000 |
| NK cell_MCPCOUNTER | MCPCOUNTER | -0.1026 | 0.0219 |
| B cell_MCPCOUNTER | MCPCOUNTER | -0.3963 | 0.0000 |
| Myeloid dendritic cell_MCPCOUNTER | MCPCOUNTER | -0.3046 | 0.0000 |
| Cancer associated fibroblast_MCPCOUNTER | MCPCOUNTER | 0.1082 | 0.0156 |
| Myeloid dendritic cell activated_XCELL | XCELL | -0.3047 | 0.0000 |
| B cell_XCELL | XCELL | -0.3889 | 0.0000 |
| T cell CD4+ naive_XCELL | XCELL | -0.2326 | 0.0000 |
| T cell CD4+ central memory_XCELL | XCELL | -0.1499 | 0.0008 |
| T cell CD8+_XCELL | XCELL | -0.3155 | 0.0000 |
| T cell CD8+ central memory_XCELL | XCELL | -0.2811 | 0.0000 |
| T cell CD8+ effector memory_XCELL | XCELL | -0.1468 | 0.0010 |
| Class-switched memory B cell_XCELL | XCELL | -0.3718 | 0.0000 |
| Common lymphoid progenitor_XCELL | XCELL | 0.1941 | 0.0000 |
| Common myeloid progenitor_XCELL | XCELL | -0.1142 | 0.0106 |
| Myeloid dendritic cell_XCELL | XCELL | -0.2603 | 0.0000 |
| Endothelial cell_XCELL | XCELL | -0.0944 | 0.0351 |
| Cancer associated fibroblast_XCELL | XCELL | -0.1988 | 0.0000 |
| Granulocyte-monocyte progenitor_XCELL | XCELL | -0.1125 | 0.0119 |
| Hematopoietic stem cell_XCELL | XCELL | -0.2075 | 0.0000 |
| Mast cell_XCELL | XCELL | -0.1150 | 0.0102 |
| B cell memory_XCELL | XCELL | -0.2553 | 0.0000 |
| B cell naive_XCELL | XCELL | -0.1256 | 0.0050 |
| Plasmacytoid dendritic cell_XCELL | XCELL | -0.2752 | 0.0000 |
| B cell plasma_XCELL | XCELL | -0.3724 | 0.0000 |
| T cell gamma delta_XCELL | XCELL | -0.2019 | 0.0000 |
| T cell CD4+ Th1_XCELL | XCELL | -0.1473 | 0.0010 |
| T cell regulatory (Tregs)_XCELL | XCELL | -0.1111 | 0.0130 |
| immune score_XCELL | XCELL | -0.3579 | 0.0000 |
| stroma score_XCELL | XCELL | -0.1848 | 0.0000 |
| microenvironment score_XCELL | XCELL | -0.3738 | 0.0000 |
| B cell_EPIC | EPIC | -0.3663 | 0.0000 |
| Cancer associated fibroblast_EPIC | EPIC | 0.1138 | 0.0110 |
| T cell CD4+_EPIC | EPIC | 0.1634 | 0.0003 |
| T cell CD8+_EPIC | EPIC | -0.2157 | 0.0000 |
| Endothelial cell_EPIC | EPIC | -0.2003 | 0.0000 |
